# Immunosuppressive effect of *Plantago major* on the innate immunity of *Galleria mellonella*

**DOI:** 10.7717/peerj.15982

**Published:** 2023-09-22

**Authors:** Serhat Kaya

**Affiliations:** Department of Biology/Faculty of Science, Çanakkale Onsekiz Mart University, Çanakkale, Turkey

**Keywords:** *Plantago major*, *Galleria mellonella*, Total hemocyte count, Cell-mediated immune responses, Humoral immune responses

## Abstract

Greater plantain (*Plantago major*), a medicinal plant species, is used in folk medicine for the treatment of various diseases in many countries of the world. Different studies have shown that the bioactive components contained in the plant have a dual effect. It was also reported that *in vivo* and *in vitro* studies showed different results. The aim of the study was to determine the effects of *P. major* extract on the hemocyte-mediated and humoral immune responses of the invertebrate model organism *Galleria mellonella*, which is widely used in immune studies. In the evaluation of these effects, total hemocyte count, encapsulation, melanization, phenoloxidase, superoxide dismutase, catalase, malondialdehyde and total protein parameters were evaluated. The results of the study showed that the total hemocyte count did not change, that the encapsulation responses decreased, that the melanization responses and phenoloxidase activity increased and that the superoxide dismutase activity decreased. As a result, it was determined that high doses of *P. major* had negative effects on cell-mediated immunity and antioxidant defence and positive effects on melanization. High doses and continuous use of *P. major* may have negative effects on living things.

## Introduction

Medicinal and aromatic plants have been indispensable resources throughout human history, both to treat diseases and to preserve/flavor their food. These plants, known as therapeutics, both serve as raw materials for some drugs and are also used in the production of biocides to protect agricultural products. Broadleaf plantain (*Plantago major*) is a therapeutic plant species. *P. major* has an increasing market demand in many European countries as well as in India, Iran, Pakistan and China (Zubair et al., 2012). It has been stated that the plant *P. major* is used for the treatment of various diseases in many different countries and that it contains both inflammatory and anti-inflammatory components, and its wound healing property may be due to the synergistic effect of not one but several components (Samuelsen, 2000). Various studies on the major effects of *P. major* on health have shown that *P. major* has antiulcerogenic (Adom et al., 2017), anti-inflammatory (Núñez Guillén et al., 1997; Hussan et al., 2015), anticancer (Chiang et al., 2003), immunomodulatory (Chiang et al., 2003), antibiotic (Najib, Alam & Halidin, 2012), antifungal (Adom et al., 2017), antiviral (Chiang et al., 2003), antioxidant (Adom et al., 2017) and diuretic (Adom et al., 2017) features.

It was found that the use of *P. major* as a feed additive caused an increase in red and white blood cells in broilers (Mazhari et al., 2016). In addition, the water extract of *P. major* was shown to have mild antiviral activities attributed to water-soluble phenolic compounds, and this study concluded that the antiviral activities were derived from the caffeic acid present in *P. major* (Chiang et al., 2002). It was determined that the *P. major* extract contained 0.05 g/ml aucubin and 0.0425 g/ml baicalein, and these substances separately showed that it inhibited the amount of reactive oxygen species (ROS) produced by human neutrophils (Reina et al., 2013). The use of *P. major* extract at a nontoxic level significantly inhibited ROS production from activated neutrophils (Reina et al., 2013). *P. major* provides protection against pneumococcal infection in mice when preadministered systemically, and this protective effect was determined to be caused by stimulation of the innate immune system (Hetland et al., 2000). Therefore, it is of great importance to determine the effects of *P. major* extract on innate immunity.

Model organisms, which are used to explain many biological phenomena, are generally determined from species whose biology is well defined and whose validity has been proven by using data obtained in similar studies (Leonelli & Ankeny, 2013). In innate immunity studies, *G. mellonella* is preferred as a model (Mylonakis et al., 2005; Mukherjee et al., 2010; Fallon et al., 2012; Ignasiak & Maxwell, 2017) because its entire genome has been determined (Lange et al., 2018), it is easy to maintain (Tsai, Loh & Proft, 2016) and is preferred in immunological studies (Hernandez et al., 2019), and because of its short life cycle (Tsai, Loh & Proft, 2016), and rapid data can be obtained (Fallon et al., 2012; Ignasiak & Maxwell, 2017). At the same time, it is a suitable subject for human pathogen studies (Cook & McArthur, 2013; Wagley et al., 2018; Trevijano-Contador & Zaragoza, 2018) since it can survive at 37 °C (Glavis-Bloom, Muhammed & Mylonakis, 2012; Champion, Wagley & Titball, 2016)). Some studies indicate that *G. mellonella* provides comparable data with other mammalian models (Champion, Wagley & Titball, 2016) and is the ideal invertebrate model organism for such studies due to the fact that its invertebrates have an immune system that is functionally similar to the innate immune system of mammals (Desbois & Coote, 2012).

Insects activate mixed immune mechanisms against these factors that endanger their bodies, such as pathogens and parasitoid/nematode eggs that enter their bodies by overcoming physical barriers (Feldhaar & Gross, 2008). Insect immune responses are classified into two main groups: humoral and hemocyte-mediated immune responses. Cellular immunity is the main defense against invading multicellular organisms such as nematodes and parasitoids (Ono et al., 2020). According to Beutler (2004), for invertebrates to mount a successful immune response, they must first recognize a pathogen by its pathogen-associated molecular pattern (PAMP) and then take appropriate measures to eliminate it by humoral and cellular effector mechanisms. Both humoral and hemocyte-mediated immune responses are related to hemocyte types in the hemolymph (Lavine & Strand, 2002).

Hemocytes in insects and myeloid cells in mammals are the cells that are functionary in cell-mediate immune response, which involves the targeting of pathogens by procedures such as phagocytosis, superoxide generation, encapsulation, and enzyme release (Sheehan et al., 2018). In the studies carried out, total hemocyte count was seen as an important marker, and an increase in hemocyte count was evaluated as boosting immunity (Altuntaş et al., 2012; Kaya et al., 2021; Kaya, Uçkan & Er, 2021; Kaya, Uçkan & Er, 2022).

As a result of the encapsulation immune response, the pathogen is inactivated by accumulating hemocytes around pathogens that are too large for hemocytes to phagocytize (such as nematodes or parasitoid eggs), isolating the pathogen. The most effective types of hemocytes in encapsulation are granulocytes and plasmatocytes (Lavine & Strand, 2002; Jiravanichpaisal, Lee & Söderhäll, 2006). Phenoloxidase (PO) levels have been shown to change in response to parasitization and affect the encapsulation rates of foreign bodies (Cotter & Wilson, 2002). The encapsulation response is the most important of hemocyte-mediated immune responses besides nodulation and phagocytosis. The encapsulation response, the most obvious manifestation of the cellular immune response, has been used by many researchers to understand innate immunity in *G. mellonella* (Ono et al., 2020; Altuntaş et al., 2012; Jiravanichpaisal, Lee & Söderhäll, 2006; Cotter & Wilson, 2002; Dubovskiy, Krukova & Glupov, 2008; Uçkan, Er & Ergin, 2010).

Among the humoral immune responses, the most effective is melanization (Lee & Anstee, 1995). The encapsulation response is often followed by the melanization response (Dubovskiy et al., 2016). Melanization completely isolates the pathogen, which is cut off from the external environment as a result of encapsulation. Melanization, the formation of a black pigment called melanin, is catalyzed by the enzyme phenoloxidase, which is converted to its active form by the serine protease cascade (Vilmos & Kurucz, 1998). Oenocytoids function during the release of PO into the hemolymph (Jiravanichpaisal, Lee & Söderhäll, 2006). Reactive oxygen species (ROS) and various metabolite reagents emerge during immune responses (encapsulation-melanization, coagulation) (Dubovskiy et al., 2016; Grizanova et al., 2018).

Many studies have examined the effects of various natural or chemical substances on the antioxidant enzymes of the model organism *G. mellonella* (Dubovskiy, Krukova & Glupov, 2008; Büyükgüzel & Kayaoğlu, 2014; Dere, Altuntaş & Nurullahoğlu, 2015; Kaya, 2022). With these studies, the effects of the agent used on *G. mellonella* oxidative stress were determined, and its physiological effects in this living organism were revealed.

The fact that *P. major* is used for wound healing (Samuelsen, 2000) suggests that it increases mitosis in the area where it is applied. However, the fact that bioactive components contained in the leaf extract have both inflammatory and anti-inflammatory properties (Samuelsen, 2000) and the mechanisms of action of these components, which have opposite effects, have not been fully demonstrated, revealing the need for more research on this plant. At the same time, a comprehensive evaluation of the mutagenic and genotoxic effects of the plant as well as its effects on humoral immunity is required.

The hypothesis of the study is that *P. major* extract has effects on innate immunity. In this study, it was aimed to determine the effects of *P. major* extract on innate immunity through the immune responses of the invertebrate model organism *G. mellonella* and to create a projection for future studies. For this purpose, in our study, encapsulation responses to determine hemocyte-mediated immune responses of the model organism *G. mellonella*, as well as PO activity and melanization response for humoral immunity, were evaluated. In addition, antioxidant enzymes were evaluated to determine oxidative stress. The most important goal of this study is to determine the effects of *P. major* extract on invertebrate model organism immunity *in vivo*. The data from this study are expected to be the starting point for evaluating the effect of this plant on all other animals. Our results showed that overdose usage of *P. major* caused a decrease in hemocyte-mediated immunity but also an increase in humoral immune responses.

## Materials & Methods

All chemicals used in this study were purchased from Sigma-AldrichSt, Louis, MO, USA.

### Insect rearing

The *G. mellonella* larvae used in this study were sourced from a successive insect culture at the Insect Physiology Research Laboratory, Faculty of Science, Çanakkale Onsekiz Mart University. The larvae were grown under controlled conditions of constant darkness, with a temperature of 28  ± 1°C and a relative humidity (RH) of 65  ± 5%. The larvae used in this study were grown under these specific conditions. Two male and four female adult *G. mellonella* moths were placed in a 1 L glass jar with 2 g of natural blackened honeycomb. When the hatched larvae were observed, the larvae were fed artificial food (natural blackened honeycomb, wheat bran, honey, water and glycerin) developed by Bronskill (1961). As the food was depleted, 10 g of artificial food, which is the average daily consumption amount, was continued to be added to the jar, and the larvae were maintained.

### Plant materials

The *P. major* samples collected from nature (UTM Region: 35 Hemisphere: N East: 445576 North: 4420312) were brought to the laboratory and were identified by Dr. Ersin Karabacak. The approved samples were prepared for extraction by being dried in a dark, well-ventilated room at room temperature. A total of 500 g of dry plant material was obtained. The dried leaves were first cut into small pieces by hand and then extracted with methanol in accordance with the study of Gomez-Flores et al. (2000). According to the additional literature reviewed, the extract was obtained by the maceration method due to the negative effect of heat extraction on *P. major* active substances (Gomez-Flores et al., 2000; Zubair et al., 2011). According to this method, the powder obtained from the leaves was placed on filter paper and placed in a one-liter glass jar. Absolute methanol (400 mL) was added to this plant powder, and extraction was performed in a dark environment for three weeks. At the end of the third week, the plant material was removed from the methanol with filter paper. Afterwards, methanol containing the extract was removed in a rotary evaporator (Omnilab, China) at 27 °C to obtain the dry matter. The dry matter obtained after the removal of the solvent was dissolved in phosphate buffered saline (PBS, pH: 7.4) at a rate of 200 mg mL^−1^. The prepared solution was injected into the subjects, and the survival status of the samples was observed for 72 h. This rate was determined as the highest dose since no death was observed in the subjects. The 80%, 60%, 50%, 40%, 30%, 20% and 10% dilutions of the highest dose were determined as experimental doses. For the control groups, untreated (positive control) and PBS (negative control)-injected samples were used.

### Injection

Last instar *G. mellonella* larvae (0.18  ± 0.02 g) were selected and used in the experiments. The selected subjects were cooled on ice and injected under a stereomicroscope (Leica EZ4, Wetzlar, Germany) *via* a 25 µl microinjector (Hamilton, Reno, NV, USA) from the last right proleg at a dose of 5 µl per larva. The larvae were kept on ice for two minutes. Experimental conditions were equalized for all samples by applying this procedure to all experimental groups, including untreated larvae.

### Total hemocyte count

The total hemocyte count was determined according to Kaya, Uçkan & Er (2021). Accordingly, a wound was made from the anterior segment of the prolegs from the injected larvae with the help of a sterile needle. Four microliters of hemolymph leaking from puncture wound was taken and placed in a microcentrifuge tube containing 36 µl of anticoagulant (0.098 M NaOH, 0.186 M NaCl, 0.017 M Na_2_EDTA and 0.041 M citric acid, pH: 4.5). Ten microliters of the prepared anticoagulant-hemolymph mixture was taken and loaded into a Neubauer improved hemocytometer (Marienfeld, Lauda-Königshofen, Germany), and cell counts were performed under a phase contrast microscope (Soptop CX41, China). The data were evaluated as ×10^6^ cells mL^−1^.

### Encapsulation-melanization

To provoke encapsulation-melanization responses, Sephadex A-25 chromatography beads stained with 1% Coomassie Brilliant Blue-G 250 dye were injected into the body cavity of larvae with an average of 15 beads in 10 µl PBS solution. Bead injection was performed from the last of the prolegs to the left. At the end of the 4th hour (short period) and 24th hour (long period) following the injection, the larvae were dissected under a stereo microscope, and the beads were collected. The collected beads were examined under a phase-contrast microscope and classified according to Richards & Dani (2008) and Kaya, Uçkan & Er (2021), and their encapsulation-melanization responses were evaluated. For an encapsulation response, if there are 0–2 rows of cell layers around the beads, it is evaluated as non-encapsulated; if there are 3–8 rows, it is weak; if there are 9 or more rows, it is evaluated as strong. According to Kaya, Uçkan & Er (2021), in the melanization response, none of the melanization on the bead was accepted as non-melanized. Specifically, up to 20% of the beads showed weak melanization, between 20% and 70% showed middle melanization, and over 70% showed strong melanization.

### Hemolymph collection

The larvae injected with experimental doses were pierced from the anterior segment of their prolegs using a suture needle after 24 h of waiting time. Thirty microliters of hemolymph leaking from the wound in the body was collected into microcentrifuge tubes containing 270 µl of phosphate buffer solution. The cell-free supernatant was obtained by centrifuging the hemolymph tenfold diluted with phosphate buffer solution for 5 min at 10,000 ×g (IKA G-L, Germany). The resulting supernatant was frozen in liquid nitrogen and stored in a −80 freezer (Panasonic, Osaka, Japan) until the enzyme activities were measured.

### Total protein

The total amount of protein from the collected hemolymph was determined according to the method of Bradford (1976). The total amount of protein in the hemolymph collected from the subjects was determined by reading the absorbance at 595 nm in a microplate reader (Multiscan FC, Thermo Scientific, Waltham, MA, USA). The Bradford curve was generated using a microplate reader prior to conducting the experiments. Using this method, 5 µl of supernatant, 155 µl of sterile distilled water, and 40 µl of Bradford reagent were added to each well of the microplate. The mixture was then incubated at room temperature for 30 min. Following this step, the absorbance value was determined using a single reading on the microplate reader.

### Phenoloxidase (PO) activity

The phenoloxidase (PO) activity was determined according to the study of Kaya (2020). As a substrate, 3 mM L-DOPA (3,4-dihydroxy-L-phenylalanine) were used. According to this method, 40 µl of the supernatant was placed in microplate wells, and 160 µl of L-DOPA was added. The enzyme activity was determined at 420 nm absorbance by placing the prepared microplate on the reader and making a total of seven measurements over a period of 30 min at five-minute intervals. The enzyme activity is given as U mg protein^−1^ min^−1^.

### Antioxidants

In our study, the method of Kaya (2022) was used. All test readings were made in the microplate reader at the appropriate wavelengths and on the microplates. The experimental results were calculated by comparing each subject’s own total protein amount.

To determine the catalase (CAT) activity in the prepared supernatant, kinetic measurements were made at 240 nm, and the change in absorbance was determined for two minutes. The data to be obtained were calculated according to Aebi (1984). The results in CAT activity were determined as mmol min^−1^ mg protein^−1^. Similarly, 9.5 µl of supernatant, 2.5 µl of Xantine oxidase and 190 µl of superoxide dismutase (SOD) reagent were added to each well of the microplate to determine the SOD activity, and the plate was incubated with light for 10 min. At the end of the time, 2.5 µl of CuCl_2_ was added to each well, and its absorbance at 560 nm was determined. The amount of SOD was calculated as unit mg protein^−1^. The Flöhe & Ötting (1984) method was used to calculate the SOD results. The formation of malondialdehyde (MDA) was measured to determine lipid peroxidation (LPO). For this, 75 µl of supernatant and 150 µl of TBA (2-thiobarbituric acid) and TCA (trichloroacetic acid) mixture were added to each well in the microplate. Afterwards, these microplates were incubated at 90 °C for 30 min, and the LPO level was determined by measuring the TBA-MDA complex spectrophotometrically at 532 nm. The data obtained were calculated according to the Buege & Aust (1978) method. The MDA content was calculated as nmol mg protein^−1^.

### Statistics

Four replicates were performed for each of the hemocyte count, encapsulation, and melanization experiments and at all doses, and four larvae were used in each replicate (*n* = 16). Two more subgroups were formed for encapsulation and melanization at the 4th hour and 24th hour. A total of 16 samples were used for each dose in each experiment. The data obtained from these samples were evaluated by one-way ANOVA (Tukey HSD) with the SPSS v20 program (*p* < 0.05). Before the ANOVA test, the normality and homogeneity test of the data were performed.

## Results

### Total hemocyte count

**Figure 1:**
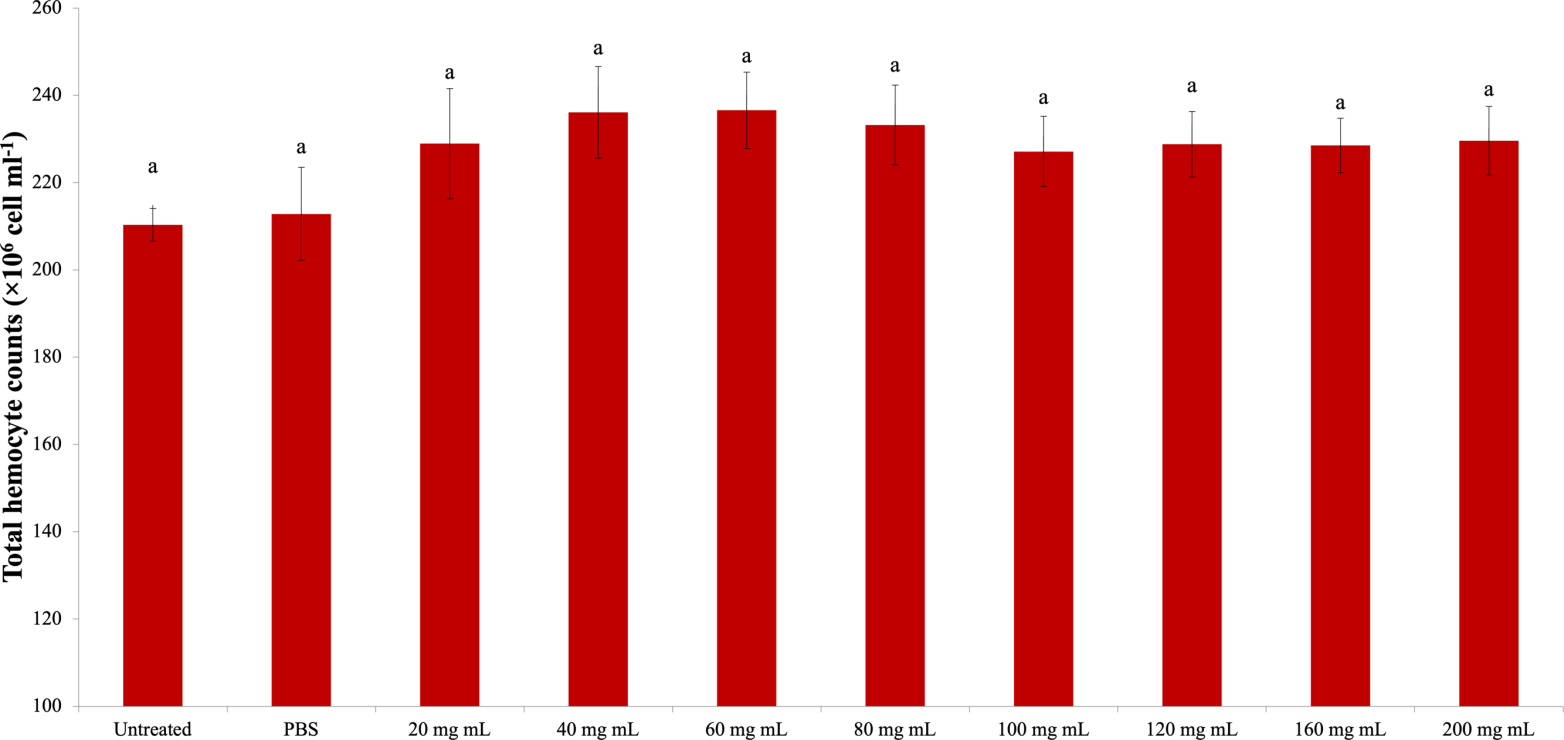
Effects of the different doses of *P. major* on the total hemocyte count in the larval hemolymh of *G. mellonella*. There was no difference between groups with the same letter (A) (*P* > 0.05). Each bar represents the mean of the 16 samples.

The change in the *G. mellonella* total hemocyte counts due to the *P. major* dose injection is presented in Fig. 1. According to the statistical evaluation, the *P. major* injection did not cause a significant change in the total hemocyte count of *G. mellonella* (dF: 9, 150; F: 1.011; p: 0.434). The highest total hemocyte count was determined in the 40 mg mL^−1^ (23.62 × 10^6^ mg mL^−1^) dose injection group, and the lowest total hemocyte count was found in the untreated group (21.03 × 10^6^ mg mL^−1^).

### Encapsulation

The changes in encapsulation responses in *G. mellonella* larvae due to the *P. major* extract injection are shown in Table 1.

**Table 1:** Effect on *G. mellonella* encapsulation response depending on *P. major* doses.

***P. major* doses**	**Total beads**	**None**	**Weak**	**Strong**
	4 h^e^ (% ± se)^f^			
Untreated	355	4.093 ± 1.346^a^	66.423 ± 3.540^a^	29.484 ± 3.714^a^
PBS	403	6.630 ± 0,996^a^	68.932 ± 1.131^ab^	24.437 ± 1.508^ab^
20 mg mL^−1^	386	18.553 ± 2,282^bc^	64.918 ± 3.278^a^	16.529 ± 2.190^bc^
40 mg mL^−1^	341	10.706 ± 2,827^ab^	75.815 ± 3.909^abc^	13.479 ± 2.724^c^
60 mg mL^−1^	391	19.249 ± 3,716^c^	68.303 ± 3.707^ab^	12.448 ± 2.633^c^
80 mg mL^−1^	360	7.154 ± 1,575^a^	80.006 ± 3.186^bc^	12.840 ± 2.630^c^
100 mg mL^−1^	372	12.575 ± 2,059^abc^	80.448 ± 2.612^bc^	6.977 ± 1.769^c^
120 mg mL^−1^	314	4.675 ± 1,291^a^	83.033 ± 2.028^c^	12.292 ± 1.967^c^
160 mg mL^−1^	405	7.419 ± 1,546^a^	75.000 ± 2.578^abc^	16.581 ± 2.595^bc^
200 mg mL^−1^	361	4.863 ± 0,833^a^	85.592 ± 2.258^c^	9.545 ± 2.464^c^
	24 h^e^ (% ± se)^f^			
Untreated	369	6.806 ± 1.220^ab^	38.382 ± 1.415^a^	54.813 ± 1.737^a^
PBS	358	2.678 ± 1.097^a^	40.859 ± 1.610^a^	56.463 ± 1.302^a^
20 mg mL^−1^	328	11.796 ± 3.004^b^	73.172 ± 6.086^b^	17.753 ± 3.879^b^
40 mg mL^−1^	332	4.812 ± 1.469^a^	75.635 ± 3.858^cd^	19.553 ± 3.937^b^
60 mg mL^−1^	355	3.645 ± 0.870^a^	75.876 ± 2.733^cd^	20.479 ± 2.469^b^
80 mg mL^−1^	314	7.930 ± 2.273^ab^	71.382 ± 3.505^bc^	20.688 ± 3.564^b^
100 mg mL^−1^	330	5.276 ± 1.558^ab^	76.267 ± 3.396^cd^	18.456 ± 3.361^b^
120 mg mL^−1^	318	3.076 ± 1.079^a^	71.490 ± 2.365^bc^	25.435 ± 2.008^b^
160 mg mL^−1^	344	6.694 ± 1.151^ab^	70.720 ± 2.476^bc^	22.586 ± 2.818^b^
200 mg mL^−1^	327	2.271 ± 0.928^a^	83.655 ± 2.720^d^	14.074 ± 2.520^b^

**Notes.**

Data are expressed as percent mean. There was no difference between groups with the same letter in the same column (*P* > 0.05). Each data point represents the mean of 16 samples.

e The short period represents 4 h and the long period 24 h data.

f Each data represents % mean ± standard error values obtained from a total of 16 larvae in four replicates.

According to the findings, the number of unencapsulated beads in the short period showed a statistically significant increase in the 20 and 60 mg mL^−1^ groups (none: df: 9, 150; F: 7.545; P: 0.00). The rate of weakly encapsulated beads increased significantly in the 120 and 200 mg mL^−1^ groups compared to the control groups (weak df: 9, 150; F: 5,994; P: 0.00). There was a significant decrease between the control groups and all dose injection groups in terms of strongly encapsulated bead rate (strong df: 9, 150; F: 7,864; P: 0.00).

According to the data obtained at the end of the long period, there was no significant difference between the control groups and the dose injection groups in terms of the number of unencapsulated beads. Nevertheless; In terms of unencapsulated beads, the difference between 20 mg mL^−1^ dose injection and PBS, 40 mg mL^−1^, 60 mg mL^−1^, 120 mg mL^−1^ and 200 mg mL^−1^ doses is significant. (none df: 9, 150; F: 3.315; P: 0.00). However, there were significant differences between the control groups and the dose injection groups in terms of both weak and strong encapsulation responses (weak df: 9, 150; F: 19,063; P: 0.00; strong df: 9, 150; F: 29,890; P: 0.00). Compared to the control groups, the number of beads with weak encapsulation increased, while the number of beads with strong encapsulation decreased. The group with the highest weak encapsulation and the lowest strong encapsulation response in the long period was 200 mg mL^−1^.

### Melanization

The results of the study regarding melanization responses are shown in Table 2. In the short period, the rate of nonmelanized beads increased in all dose injection groups compared to the control groups. There was no difference between the groups in terms of weak melanization responses in beads with melanization. However, there was a significant difference between the control groups and the dose injection groups and between the moderate and strong elanization groups. (4 h none: df: 9, 150; F: 11.141; P: 0.00; weak df: 9, 150; F: 1.351; P: 0.226; middle df: 9, 150; F: 7.524; P: 0.00; strong df: 9, 150; F: 13.625; P: 0.00)

**Table 2:** Effect of *G. mellonella* on melanization response depending on *P. major* doses.

***P. major* doses**	**None**	**Weak**	**Middle**	**Strong**
	4 h^e^ (% ± se)^f^			
Untreated	18.024 ± 2.916^a^	20.619 ± 3.922^a^	29.950 ± 3.424^a^	31.408 ± 4.225^a^
PBS	19.892 ± 1.111^a^	21.907 ± 1.351^a^	31.215 ± 0.954^a^	26.986 ± 1.891^ac^
20 mg mL^−1^	54.044 ± 4.230^bc^	22.607 ± 2.660^a^	14.506 ± 2.432^b^	8.844 ± 1.990^b^
40 mg mL^−1^	46.706 ± 4.257^bc^	28.347 ± 3.425^a^	17.383 ± 3.598^b^	7.563 ± 1.869^b^
60 mg mL^−1^	58.361 ± 4.804^b^	21.052 ± 3.281^a^	14.789 ± 2.296^b^	5.798 ± 1.787^b^
80 mg mL^−1^	51.039 ± 3.561^bc^	22.044 ± 3.393^a^	20.042 ± 2.059^b^	6.875 ± 1.524^b^
100 mg mL^−1^	53.261 ± 4.986^bc^	26.171 ± 4.433^a^	14.841 ± 2.223^b^	5.727 ± 1.873^b^
120 mg mL^−1^	46.383 ± 4.806^bc^	28.689 ± 2.982^a^	14.266 ± 2.511^b^	10.662 ± 1.881^b^
160 mg mL^−1^	41.477 ± 3.371^c^	19.967 ± 2.683^a^	18.760 ± 2.122^b^	19.796 ± 4.258^c^
200 mg mL^−1^	50.502 ± 3.036^bc^	29.761 ± 2.717^a^	11.699 ± 1.538^b^	8.038 ± 1.060^b^
	24 h^e^ (% ± se)^f^			
Untreated	19.658 ± 3.325^a^	14.754 ± 1.276^a^	26.392 ± 1.925^ab^	39.196 ± 2.786^a^
PBS	19.699 ± 1.777^a^	15.198 ± 2.751^a^	27.682 ± 1.882^ab^	37.421 ± 2.735^a^
20 mg mL^−1^	9.124 ± 0.945^b^	32.123 ± 2.784^bc^	25.469 ± 1.816^ab^	33.284 ± 2.692^a^
40 mg mL^−1^	7.974 ± 1.343^b^	32.662 ± 3.759^bc^	35.094 ± 3.953^a^	24.270 ± 6.527^a^
60 mg mL^−1^	7.237 ± 1.391^b^	35.517 ± 3.041^bc^	24.436 ± 2.838^ab^	32.809 ± 4.578^a^
80 mg mL^−1^	7.858 ± 1.301^b^	33.057 ± 3.111^bc^	22.246 ± 2.166^b^	36.839 ± 4.467^a^
100 mg mL^−1^	8.454 ± 1.310^b^	37.048 ± 4.287^c^	22.486 ± 2.744^b^	32.013 ± 4.979^a^
120 mg mL^−1^	9.888 ± 2.112^b^	22.764 ± 3.113^ab^	30.168 ± 2.015^ab^	37.180 ± 4.208^a^
160 mg mL^−1^	8.781 ± 0.983^b^	27.609 ± 3.462^ab^	28.150 ± 2.484^ab^	35.461 ± 4.498^a^
200 mg mL^−1^	7.597 ± 1.943^b^	31.787 ± 2.666^bc^	29.786 ± 2.672^ab^	30.830 ± 3.900^a^

**Notes.**

Data are expressed as percent mean. There was no difference between groups with the same letter in the same column (*P* > 0.05). Each data point represents the mean of 16 samples.

e The short period represents 4 h and the long period 24 h data.

f Each data represents % mean ± standard error values obtained from a total of 16 larvae in four replicates.

According to the long-term results, the number of unmelanized beads decreased significantly in all dose injection groups compared to the control groups. The weak melanization rate increased in all dose groups compared to the control groups. There was no difference between the control groups and the other groups in terms of moderate and strong melanization responses. (24 h none df: 9, 150; F: 7.402; P: 0.00; weak df: 9, 150; F: 6.526; P: 0.00; middle df: 9, 150; F: 2.440; P: 0.013; strong df: 9, 150; F: 0.544; P: 0.841).

### Total protein

**Figure 2:**
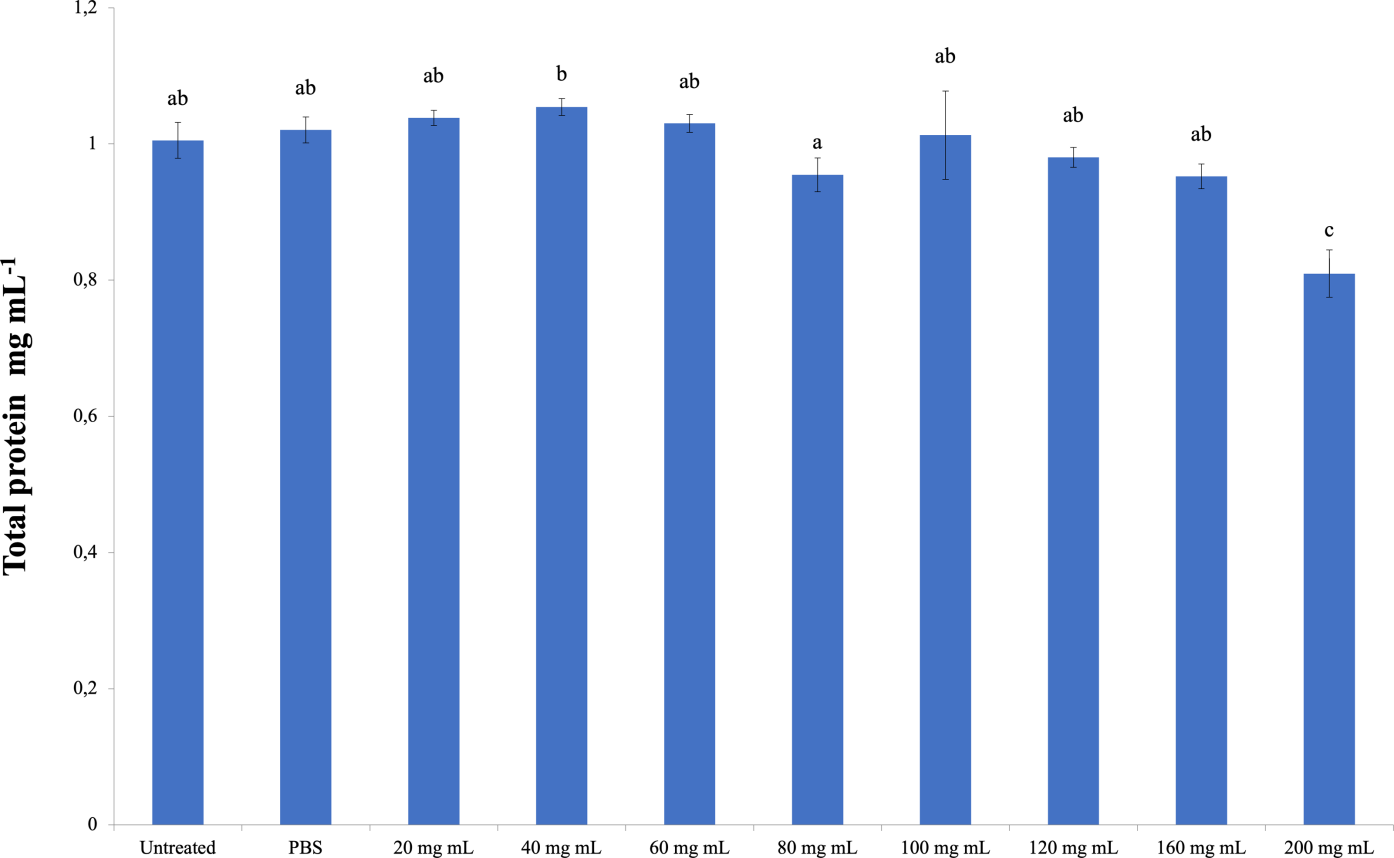
Changes in total protein amount in the hemolymh of *G. mellonella* larvae exposed to *P. major* doses. There was no difference between groups with the same letter (A–C) (*P* > 0.05). Each bar represents the mean of the 16 samples.

The total amount of hemolymph protein is presented in Fig. 2. According to the data obtained, the highest total protein amount was determined in the 40 mg mL^−1^ injection group (1.054 mg mL^−1^). The lowest total protein amount was determined in the 200 mg mL^−1^ injection group (0.810 mg mL^−1^), and the differences between that group and the control groups were significant. A significant difference was found between the 200 mg mL^−1^ group and all other groups from the injected groups. The difference between the dose injection groups except 200 mg mL^−1^ and the control groups was insignificant. At the same time, the difference between the 40 mg mL^−1^ group and the 80 mg mL^−1^ group was found to be significant (dF: 9, 150; F: 12.78; p: 0.00).

### Phenoloxidase (PO)

**Figure 3:**
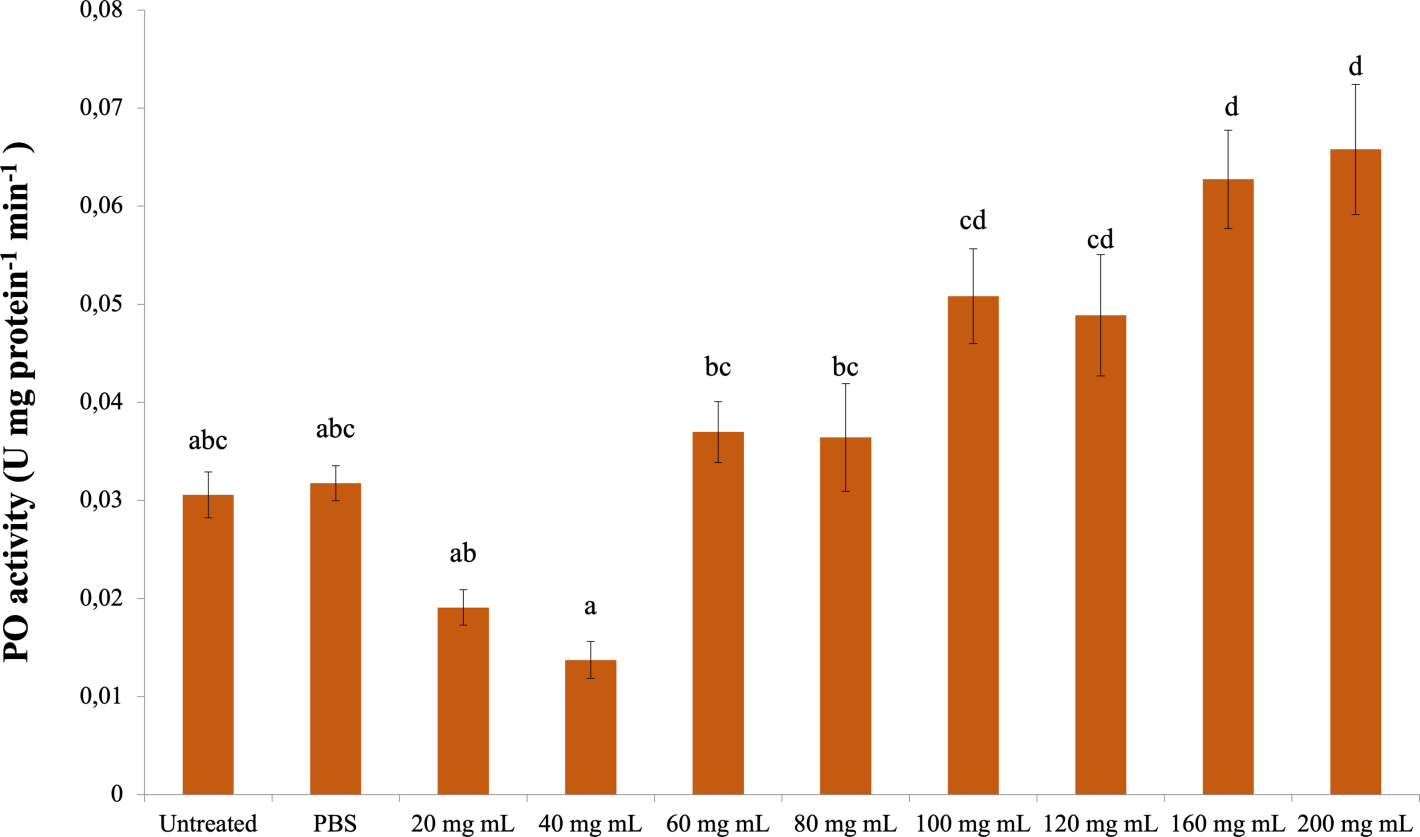
Minute changes in PO activities in the larval hemolymh of *G. mellonella* exposed to different doses of *P. major*. There was no difference between groups with the same letter (A–D) (*P* > 0.05). Each bar represents the mean of the 16 samples.

The change in PO activity is presented in Fig. 3. Accordingly, there was a significant difference between the control groups and the 160 and 200 mg mL^−1^ groups (dF: 9, 150; F: 12.57; p: 0.00); however, there was no difference between them and the other dose injection groups. At the same time, the difference between the 160 and 200 mg mL^−1^ groups and the 80 mg mL^−1^ and below injection groups was also significant ( *p* < 0.05).

### CAT

**Figure 4:**
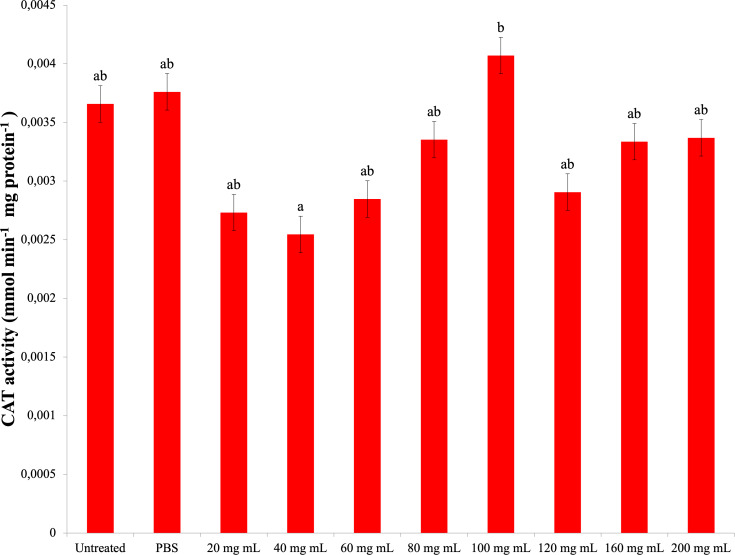
Changes activities in the larval hemolymh of *G. mellonella* exposed to different doses of *P. major.*. There was no difference between groups with the same letter (A–B) (*P* > 0.05). Each bar represents the mean of the 16 samples.

As a result of experiments to determine catalase enzyme activity, no significant difference was found between the *P. major* injection groups and the control groups. The highest CAT activity was determined in the 100 mg mL^−1^ dose injection group (0.004 mmol min^−1^ mg protein^−1^) (Fig. 4). The difference between the *P. major* injection groups and their own 40 mg mL^−1^ and 100 mg mL^−1^ groups was significant (dF: 9 150; F: 2.228; p: 0.023). The difference between the other groups and these groups was insignificant (*p* > 0.05).

### SOD

**Figure 5:**
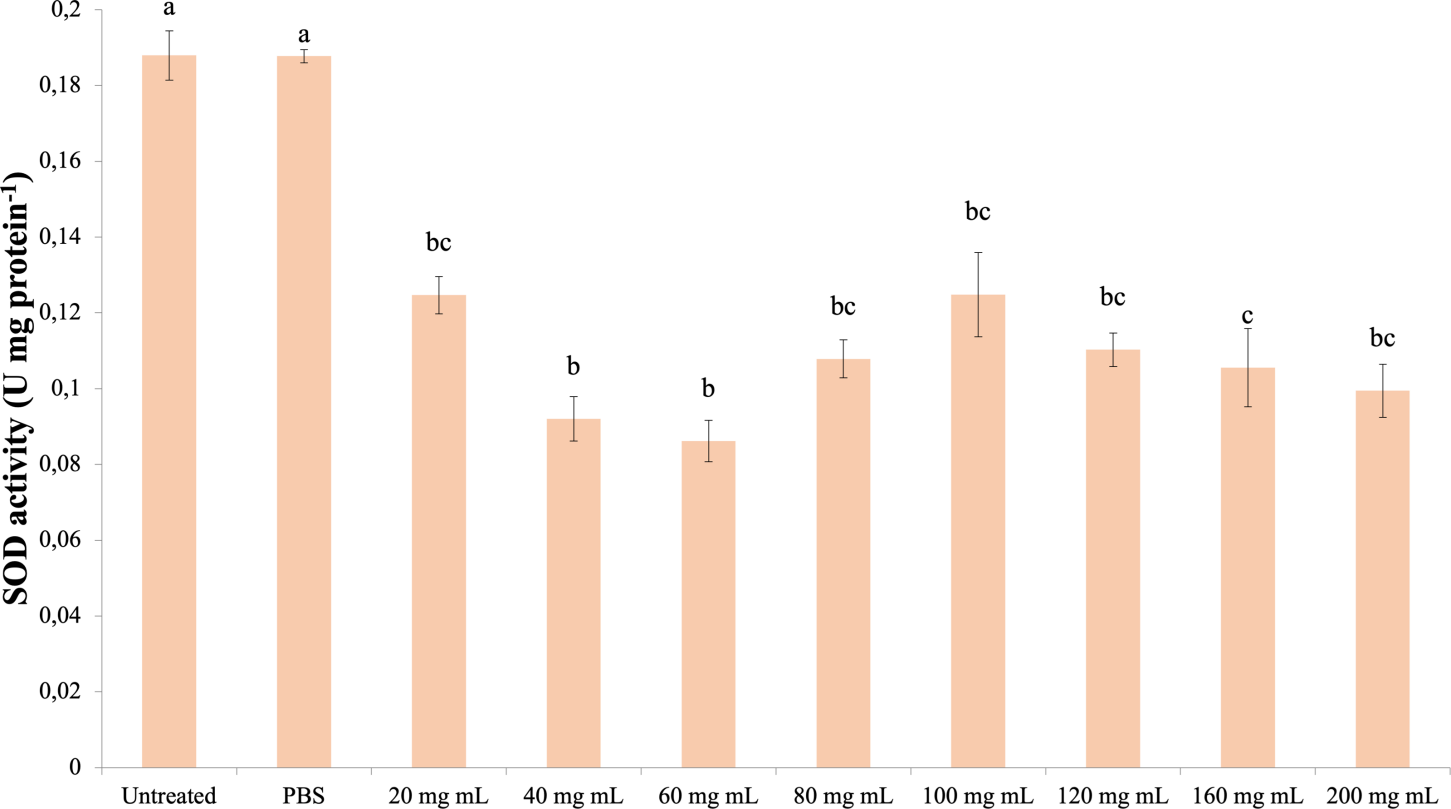
Effects of the *P. major* doses on the SOD activity in the hemolymph of *G. mellonella* larvae. There was no difference between groups with the same letter (A–C) (*P* > 0.05). Each bar represents the mean of the 16 samples.

The data obtained as a result of the experiments in which the superoxide dismutase activity was determined are presented in Fig. 5. Accordingly, all dose injections showed significantly lower SOD activity compared to the control groups (dF: 9, 150; F: 15,798; p: 0.00). The lowest SOD activity was determined in the 60 mg mL^−1^ group. There was also a significant difference between the injection groups 40 mg mL^−1^ and 60 mg mL^−1^ and the 160 mg mL^−1^ group. The difference between the other injection groups and these groups and among themselves was insignificant (*p* > 0.05).

### MDA

**Figure 6:**
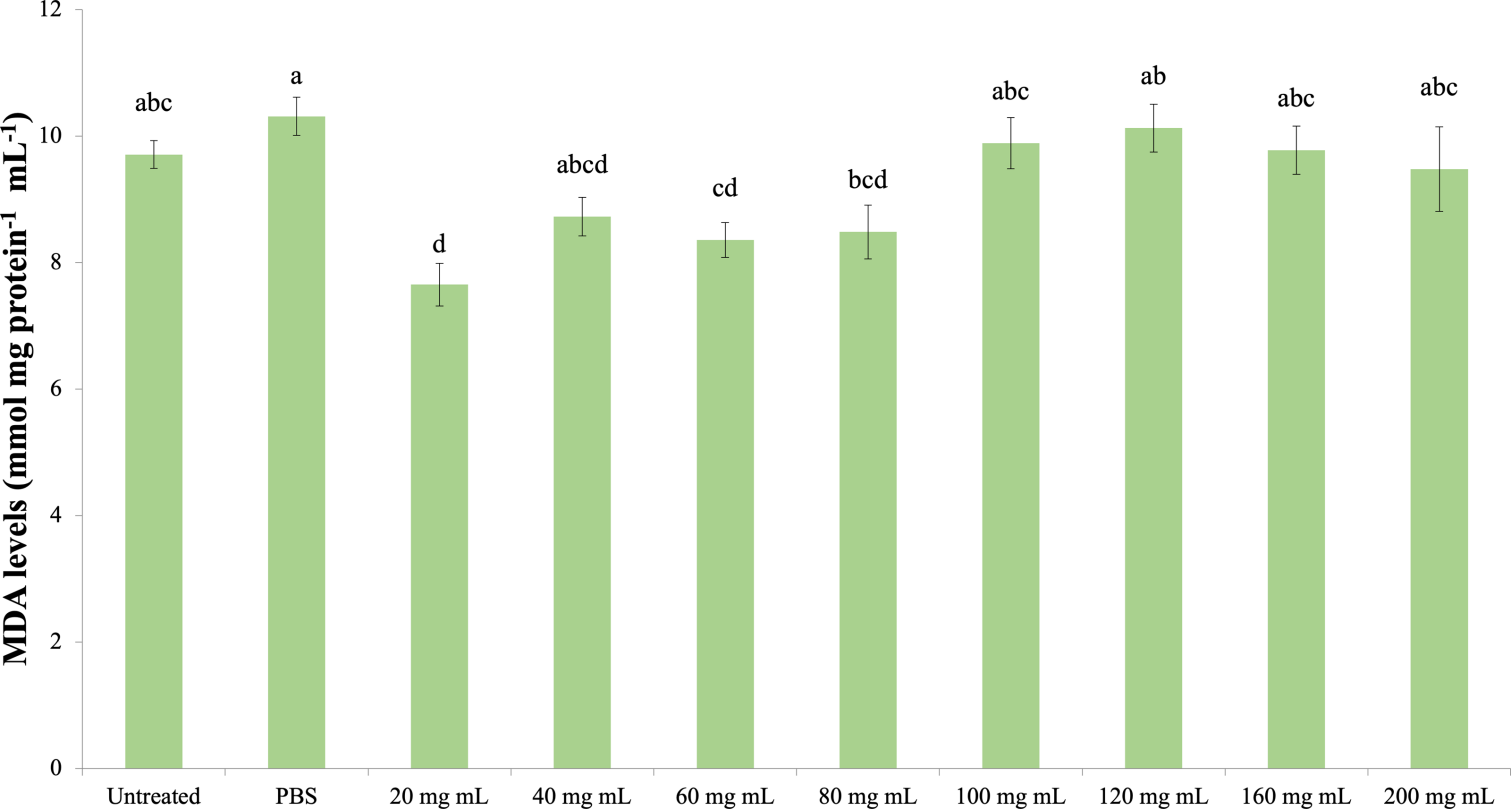
Effects of the *P. major* doses on the MDA amount in larval haemolymph of *G. mellonella*. There was no difference between groups with the same letter (A–D) (*P* > 0.05). Each bar represents the mean of the 16 samples.

The effect of the *P. major* injection on the amount of MDA in *G. mellonella* hemolymph is presented in Fig. 6 (dF: 9, 150; F: 12.535; p: 0.00). Accordingly, the lowest MDA amount was determined in the 20 mg mL^−1^ group. A significant difference was determined between the control groups and the 20 mg mL^−1^ group (*p* < 0.05). At the same time, the difference between the 20 mg mL^−1^ group and the 40, 60 and 80 mg mL^−1^ groups was insignificant (*p* > 0.05), while the difference between the other groups (100 mg mL^−1^ and above doses) was significant (*p* < 0.05).

## Discussion

According to the results of the study, *P. major* leaf extract did not cause any change in the total hemocyte count of *G. mellonella*. However, it reduced the strong encapsulation response in both the short and long periods compared to the control groups. On melanization from humoral immune responses, the number of nonmelanized beads in the short period increased compared to the control groups. In the long period, the number of non-melanized beads decreased compared to the control groups. The PO activity also coincided with the long-term results of melanization and differed significantly from the control at doses of 160–200 mg mL^−1^.

It was also determined that the *P. major* leaf extract decreased the amount of TP in *G. mellonella* hemolymph at a dose of 200 mg mL^−1^; however, it did not cause any change in the CAT activity. In terms of SOD activity, all doses of *P. major* leaf extract showed a negative effect compared to the control groups. There was a decrease in MDA formation only at the 20 mg mL^−1^ dose compared to the control groups.

These results showed that the *P. major* leaf extract did not change the *G. mellonella* hemocyte count but changed their behavior and increased humoral immunity in the long term. In addition, it was thought that the antioxidant enzymes had a negative effect on SOD activity and that with these aspects, the *P. major* leaf extract played a critical role in immunity.

While the effects of the plant extracts on the total hemocyte count or lysis activity were different between *in vitro* and *in vivo*, the PO activity and its effects on the phagocytic activity showed a strong correlation in *in vitro* and *in vivo* experimental results (Huang et al., 2018). For this reason, examining the effects of plant extracts on immunity by *in vivo* studies with model organisms provides healthier results.

It was determined that the highest concentration (10 mg/ml) of ethanol-based *P. major* extract with high polyphenol content prevented cell proliferation and migration (Zubair et al., 2012). This effect on cell proliferation suggests the need for careful optimization of dosages for treatments. The total hemocyte count results of our study showed that the *P. major* extract did not cause a significant change in hemocyte count depending on the dose. The result of our study regarding the total hemocyte count of the *P. major* extract is consistent with the results of Zubair et al. (2012) and Huang et al. (2018).

In a study with the calyx fluid of *Pimpla turionellae*, a member of the hymenoptera living as a pupal parasitoid in *G. mellonela*, it was determined that the application of the calyx reduced the encapsulation response at all doses (Kaya, Uçkan & Er, 2022). Uçkan, Er & Ergin (2010) determined that it reduced the encapsulation response in *G. mellonella* with *P. turionellae* venom. It was determined that gibberellic acid (GA_3_) application caused a decrease in encapsulation-melanization responses in *G. mellonella* (Altuntaş et al., 2012). In a study examining the effect of boric acid (BA) on the encapsulation response of *G. mellonella*, it was determined that it caused a decrease in encapsulation and melanization responses (Gwokyalya & Altuntaş, 2019). According to Kaya & Demir (2020), olive leaf extract increased encapsulation and melanisation responses significantly at a 1,000 ppm dose compared to the control group in *G. mellonella*. It was determined that the application of *Helichrysum arenarium* extract significantly increased the encapsulation and melanisation response in *G. mellonella* at doses of 0.25 and 0.50% in 24 h compared to the control group (Kaya et al., 2021). Indole-3-acetic acid (IAA) application at a dose of 50 ppm increased the number of unencapsulated beads in the encapsulation response but did not change the weak and strong encapsulation responses in *G. mellonella* (Kaya, Uçkan & Er, 2021). It was determined that the application of ethephon, a plant growth regulator, suppressed the encapsulation response in *G. mellonella* (Altuntaş, Gwokyalya & Bayram, 2022). As can be understood from the literature, *G. mellonella* encapsulation and melanization immune responses were evaluated together. Plant secondary metabolites and plant growth regulators appear to reduce the encapsulation and melanization response at high doses. It is thought that *P. major* extract causes a decrease in the encapsulation response and prevents granulocytes from attracting plasmatocytes to this region by binding to the invading surface. Zubair et al. (2012) reported that high doses of *P. major* extract inhibited cell migration. Encapsulation requires hemocytes to adhere to the invader and, in a sense, to migrate, and inhibiting this reduces the encapsulation response. Our encapsulation results are in accordance with the studies of Zubair et al. (2012). Our results support each other with the result of decreased cell proliferation at high doses.

Studies in which the effects of plant extracts on *G. mellonella* hemolymph phenoloxidase (PO) activity were determined show that enzyme activity varies depending on the extract dose. Kaya et al. (2021) determined in their study that *H. arenarium* extract did not change PO activity at the lowest and highest doses in *G. mellonella* compared to the control groups but increased PO activity at the intermediate doses. It was determined that the pyrethrum extract obtained from *Chrysanthemum cinerariaefolium*, which has a strong toxic effect on insects, increased the PO activity of *G. mellonella* hemolymph at a dose of 0.6 mg mL^−1^ compared to the control but did not affect it at higher doses (Kaya, 2020). It was demonstrated that *Olea europaea* leaf extract increased the PO activity of *G. mellonella* hemolymph at the lowest dose used in the study (0.001 mg mL^−1^) compared to the control group but did not affect it at higher doses (Kaya & Türkdoğan, 2021). As a result of IAA administration, it was determined that *G. mellonella* hemolymph was significantly decreased at all doses compared to the control group (Aksan, Uçkan & Er, 2022). In the results of our study, the PO activity increased significantly at 160 and 200 mg mL^−1^ doses compared to the control groups. Previous studies have shown that plant extracts increase PO activity at certain doses. In our study, the increase in PO activity at the highest doses and the gradual change at other doses means that the humoral immune response varies depending on the dose of *P. major* extract.

Studies with different plant extracts show that plant phenolic contents provide the expected benefit on the model organism antioxidant defense system at low doses.

In a study examining the effects of guava (*Psidium guajava*) leaf extract on immune responses, growth performance and resistance to *Vibrio parahaemolyticus* in white shrimp (*Penaeus vannamei*), it was determined that the leaf extract enhanced the nonspecific immune response (Dewi et al., 2021). In a study conducted with *Spodoptera litura* larvae, it was determined that *Manihot esculenta* extract caused a significant increase in total and different hemocyte counts, and detoxification enzymes, such as PO, CytP450, and GST, and catalase activities significantly increased, while SOD activity decreased compared to the control group (Manjula et al., 2020). Essential oils from *Callistemon viminalis* and *Ferula gummosa* were shown to cause a drastic reduction in total hemocyte count in *Ephestia kuehniella* larvae treated in a dose-dependent manner, and plasmatocytes and granulocytes were also shown to be the most susceptible hemocytes of *E. kuehniella* larvae (Ghasemi et al., 2014). It was determined that the extract from *Dodonaea viscosa* (Dodenya) reduced the total hemocyte count of *Spodoptera exigua* (Ramírez-Zamora et al., 2020).

Studies on *G. mellonella* antioxidant enzyme activity of plant growth regulators and their secondary metabolites show that they cause changes in these activities. In the study of Dere, Altuntaş & Nurullahoğlu (2015), increasing doses of azadiractinin (AZA) decreased SOD and CAT activity and increased the amount of MDA at high doses in *G. mellonella* larvae. In another study with GA_3_, it was determined that SOD and glutathione S transferase (GST) levels increased at low doses, while CAT activity increased at all doses (Altuntaş, 2015). In a study conducted with the plant growth regulator IAA of the auxin group, it was found that IAA at doses of 500 ppm and above caused a significant decrease in SOD, CAT and GST levels compared to the control group and showed a significant increase (Özyılmaz et al., 2019). Niclosamide was shown to reduce the antioxidant enzyme activity of *G. mellonella* (Büyükgüzel & Kayaoğlu, 2014). These studies indicate that plant growth regulators and secondary metabolites reduce antioxidant enzyme activity at high doses. According to a study with *Tymbra capitata* extract, the extract reduced SOD and CAT activity in *G. mellonella* hemolymph (Kaya, 2022). The results of our study showed that the *P. major* extract did not cause a significant change in the CAT activity and MDA amount at high doses compared to the control groups. In addition, it decreased SOD activity at all doses. These results indicate that a high amount of *P. major* extract causes a negative effect on antioxidant enzymes. These results are in line with the results of studies with plant extracts and plant growth regulators. The decrease in SOD activity with no change in CAT activity seen in the results of our study is consistent with the results of Manjula et al.’s study (Manjula et al., 2020). This may be due to the bidirectional effect of *P. major* content.

It was shown that the secretion of IFN- *γ* (Interferon Gamma) and increased lymphocyte proliferation by aucubin, one of the *P. major* active substances, may be the cause of its anticancer properties (Scott & Krauss, 2012). In addition, it was determined that the methanolic extract of *P. major* leaf increased the production of nitric oxide and TNF- *α* by macrophages and stimulated lymphocyte proliferation (Gomez-Flores et al., 2000). In a study conducted with aqueous extracts of *P. major* and *P. asiatica* species, it was determined that the immunodulatory activity had dual effects, which increased lymphocyte proliferation and interferon secretion at low concentrations (<50 µg/ml) but inhibited this effect at high concentrations (>50 µg/ml) (Chiang et al., 2003). In the same study, it was stated that the aqueous extract of *P. major* had an immunomodulatory effect (Chiang et al., 2003). However, our results showed that the *P. major* extract reduced cell-mediated immunity. Other studies in the literature also confirm that *P. major* extract has adverse effects on the cell-mediated immune response (Zubair et al., 2012). This difference may be because the study of Chiang et al. (2003) was conducted with hot water extract. Some studies in the literature show that the active content of *P. major* is deteriorated by heat (Gomez-Flores et al., 2000; Zubair et al., 2011).

## Conclusions

The results of our study showed that the *P. major* extract had a dual effect. As a result, it was determined that the *P. major* extract decreased the cell-mediated immune response and increased humoral immunity at all doses in *G. mellonella*. When this information is evaluated together with the results of antioxidant activity, it is thought that excessive consumption of *P. major* will prevent cell-mediated immunity contrary to expectations and make it vulnerable to damage by oxygen radicals by lowering antioxidant enzyme activity. These results suggest that the use of *P. major* will have negative effects on immunity and may not provide the expected benefit at higher doses. Our results also show that molecular biological studies are needed to fully determine the immunosuppressive effects of *P. major* active substances. Accordingly, in future studies, it is thought that it will be essential to determine the active substances that cause this *P. major* to have a dual effect and to determine the effects of these substances on antimicrobial peptide gene expressions by using various pathogens, especially in order to determine the effects of these substances on humoral immunity. At the same time, the results obtained from this study show that this plant extract can also be evaluated as an insecticide. With future research, it is possible to determine the insecticidal compounds in this plant extract and to obtain natural compounds that can be used in integrated pest management by using them.

##  Supplemental Information

10.7717/peerj.15982/supp-1 Supplemental Information 1 Encapsulation data

10.7717/peerj.15982/supp-2 Supplemental Information 2 Melanization Data

10.7717/peerj.15982/supp-3 Supplemental Information 3 Total Hemocyte Count Data

10.7717/peerj.15982/supp-4 Supplemental Information 4 The Enzymes Data

## Additional Information and Declarations

### Data Availability

The following information was supplied regarding data availability: The data is available at Zenodo: Kaya, Serhat. (2023). Plantago major effects on the innate immunity of *Galleria mellonella* (Draft) [Data set]. Zenodo. https://doi.org/10.5281/zenodo.8167816.
